# An Approach to Integrated Scheduling of Flexible Job-Shop Considering Conflict-Free Routing Problems

**DOI:** 10.3390/s23094526

**Published:** 2023-05-06

**Authors:** Jiachen Sun, Zifeng Xu, Zhenhao Yan, Lilan Liu, Yixiang Zhang

**Affiliations:** Shanghai Key Laboratory of Intelligent Manufacturing and Robotics, School of Mechatronic Engineering and Automation, Shanghai University, Shanghai 200444, China; jiachensun1997@163.com (J.S.); xuzifeng@shu.edu.cn (Z.X.); zh_yan4869@163.com (Z.Y.); archezhang@foxmail.com (Y.Z.)

**Keywords:** integrated scheduling, flexible job-shop, conflict-free routing problem, hybrid genetic algorithm, production scheduling, automated guided vehicle, path planning

## Abstract

This study proposes an approach to minimize the maximum makespan of the integrated scheduling problem in flexible job-shop environments, taking into account conflict-free routing problems. A hybrid genetic algorithm is developed for production scheduling, and the optimal ranges of crossover and mutation probabilities are also discussed. The study applies the proposed algorithm to 82 test problems and demonstrates its superior performance over the Sliding Time Window (STW) heuristic proposed by Bilge and the Genetic Algorithm proposed by Ulusoy (UGA). For conflict-free routing problems of Automated Guided Vehicles (AGVs), the genetic algorithm based on AGV coding is used to study the AGV scheduling problem, and specific solutions are proposed to solve different conflicts. In addition, sensors on the AGVs provide real-time data to ensure that the AGVs can navigate through the environment safely and efficiently without causing any conflicts or collisions with other AGVs or objects in the environment. The Dijkstra algorithm based on a time window is used to calculate the shortest paths for all AGVs. Empirical evidence on the feasibility of the proposed approach is presented in a study of a real flexible job-shop. This approach can provide a highly efficient and accurate scheduling method for manufacturing enterprises.

## 1. Introduction

Job-shop Scheduling Problems (JSPs) are known as typical NP-complete problems [1]. Flexible Job-shop Scheduling Problems (FJSPs) are an extension of JSP. Unlike the classical JSP, the FJSP does not predefine the machine for each operation. Instead, FJSP provides the opportunity to select from multiple machines for each operation, making it more closely aligned with real-world situations [2]. The integrated scheduling problem in a flexible job shop mainly involves scheduling production machines and AGVs. It requires a scientific and reasonable scheduling arrangement for production tasks to achieve the optimal allocation of equipment and resources in the system and ultimately achieve the purpose of improving production efficiency [3].

The literature review in this paper is carried out in two aspects: the scheduling problem of flexible job-shops and the routing problem of multiple AGVs.

Ulusoy et al. [4] were the first to propose an algorithm for integrated scheduling by using AGVs and production equipment simultaneously as the object of study for the scheduling problem under the assumption that there is no path conflict. They used a genetic algorithm to solve the problem of scheduling production equipment and AGVs simultaneously and established classical cases. Subbaiah [5] used a flock genetic algorithm to solve the scheduling problem with a fixed number of AGVs. He used maximum completion time and minimum average delay as the objective function and determined the optimal solution with a smaller number of iterations. Badakhshian [6] used a fuzzy logic controller to control the operators of the genetic algorithm to filter better combinations of initial parameters. Reddy et al. [7] dealt with machine, AGV, and tool simultaneous scheduling in a multi-machine flexible manufacturing system considering jobs’ transport times among machines to minimize makespan. Erol [8] proposed a multi-agent system for simultaneous dynamic scheduling of AGVs and production equipment and compared it with five different algorithms. Zhang et al. [9] proposed an effective genetic algorithm to minimize makespan and designed Global Selection (GS) and Local Selection (LS) to generate a high-quality initial population in the initialization stage. Yan et al. [10] addressed the constraint influence imposed by finite transportation conditions in the FJSP and analyzed the coupling relationship between transportation and processing stages. Palacios [11] proposed an effective genetic algorithm hybridized with tabu search and heuristic seeding to minimize the makespan. However, the above research studies have primarily centered on production and AGV scheduling but have neglected vehicle collisions and routing problems.

Conflict-free Routing Problem (CFRP) is a policy that plans a path for an AGV to avoid path conflicts caused by paths occupied by other AGVs during its run time. These multiple AGV systems need to consider conflict avoidance and efficiency between multiple AGVs. Path planning for multiple AGVs has long been proven to be an “NP-complete” problem.

Path planning is generally divided into two stages: (1) representing the environment discretely; (2) searching for the optimal path using the graph search algorithm. In terms of discrete representation of the environment, there are two main methods: the raster method and the road map method. In most industrial scenarios, there are pre-planned routes, and road maps exist naturally. Path search is based on the road map. There are many classical algorithms for graph-based searches, such as the Dijkstra algorithm, A* algorithm, and Floyd algorithm [12]. In actual engineering, the layout of the workshop is mostly single-lane. If path planning of AGVs only considers the shortest path, AGVs are bound to encounter path conflicts during transportation. Mac T T et al. [13] used the Dijkstra algorithm to obtain an optimal conflict-free path a the triangular decomposition graph and used a genetic algorithm to smooth the path obtained from the planning. Zhang et al. [14] proposed a Dijkstra-based path planning algorithm to generate optimal collision-free paths for substation inspection multi-robots. Sun et al. [15] proposed an improved A* algorithm to solve the multi-robot collaborative path planning for the large area coverage problem. They optimized the previously calculated paths by the A* algorithm to minimize the distance traveled by the vehicle and provided the optimal solution based on the path planning problem in the multi-robot system. A deadlock is a state of a system where two or more processes are unable to proceed further because each process is waiting for the other to release the resources they need. This results in a standstill where both processes are stuck and cannot proceed unless one of the processes releases its resources. Möhring et al. [16] used a time window function to solve the common deadlock problem during conflict-free path planning. Desauliners et al. [17] proposed an exact solution to the real-time scheduling and conflict-free routing problem of AGVs, with the goal of reducing the production costs associated with production delays. Lin et al. [18] proposed a path-planning method using A* algorithm with RFID technology. This method mainly calculates the shortest path based on the coordinate information provided by the RFID tag and selects the path with the smallest angle in the current driving direction, and finally attains the result of fewer turns and the shortest path. Xing et al. [19] proposed a novel tabu search algorithm that aims to enhance the operational efficiency of AGVs in an automated warehouse. Murakami [20] addressed an AGV routing problem by formulating the dispatch and conflict-free routing problem of a capacitated AGV system as a mixed-integer linear programming problem. Yuan et al. [21] proposed a bi-level path planning algorithm to optimize the routing of multi-AGVs. Previous studies primarily focused on the vehicle routing problem and collision-free routing problem while neglecting problems of production scheduling or assuming optimality in the scheduling problem. However, optimal path planning may not necessarily align with optimal scheduling and may not result in the ideal makespan.

Most of the research in this area concentrates on production scheduling problems, AGV scheduling problems, or a combination of both. Little research has also been conducted considering the path conflicts of AGVs. Saidi-Mehrabad et al. [22] proposed an ant colony algorithm to address a JSP by considering the CFRP and the transportation time. Fu et al. [23] studied a production scheduling and vehicle routing problem with job splitting and delivery time windows in a company working in the metal packaging industry. However, their research did not specifically focus on the FJSP problem. Hence, this paper takes into account the integrated scheduling of the flexible job-shop, which involves scheduling machines and AGVs simultaneously while also considering path conflicts and transportation time.

## 2. Problem Description

### 2.1. Integrated Scheduling Problem of Flexible Job-Shop

Flexible job-shop mainly completes the manufacturing process of workpieces. As the main equipment for material transportation, AGVs complete the transportation of materials between various stations. Materials are loaded or unloaded at different stations. Therefore, AGV trips can be classified as either deadheading or loaded trips. During deadheading trips, the AGV moves empty from its current station to a different station where it loads material. In contrast, during loaded trips, the AGV moves material between stations. 

Due to the conflict routing problem, the scheduling of AGVs cannot take the shortest path but the shortest transportation time as the optimization goal. In a flexible job-shop, manufacturing machines and transportation equipment can only effectively improve the overall production efficiency by cooperating with each other. Since the conflict-free routing problem causes uncertainty in the transportation time of multiple AGVs, it is important for the optimal allocation of production resources to realize the integrated scheduling of machines and AGVs.

In the flexible job-shop, it is assumed that there are m sets of machines and w sets of AGVs, which have the same transport capacity, assigned the task of processing n workpieces. Each workpiece comprises multiple processing operations. Machines in the job-shop have a high degree of automation, and each machine can complete multiple different operations on different workpieces. Therefore, each operation can be completed by different machines. However, the time required to complete the same operation varies for different machines.

**Figure 1:**
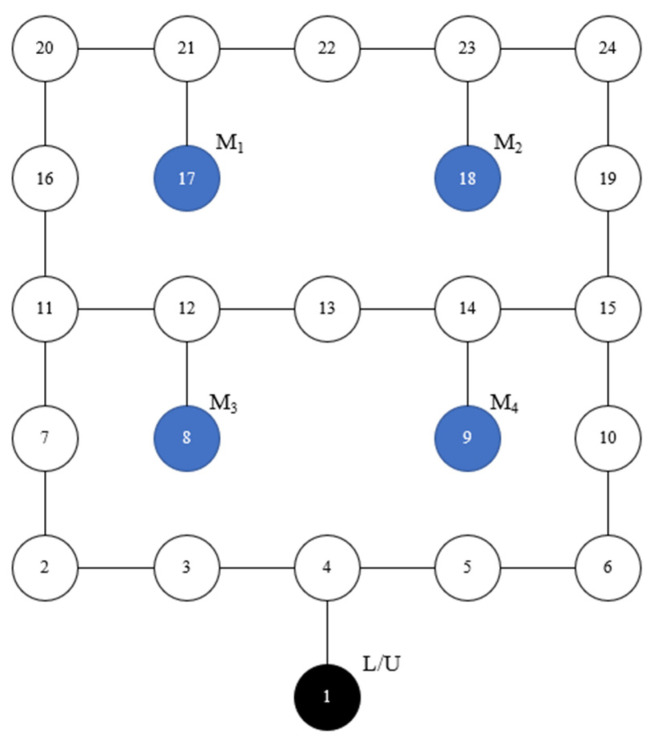
Layout of the flexible job-shop.

Our laboratory functions as a workshop for the production of personalized custom cars, with five unique models available. With each model having a unique set of operations, the predetermined durations for each operation are known. It is in line with the flexible job-shop problem. Figure 1 shows the layout of our laboratory. There are three AGVs. Node 1 is the L/U station. AGVs depart from the L/U station with the materials and carry the finished workpiece back to the L/U station. Four machines are distributed in Nodes 8, 9, 17, and 18, respectively.

Table 1 shows the processing machines for each operation and the time required. Table 2 shows the transportation time.

### 2.2. CFRP

In the study of path planning, due to the constant travel speed of AGVs, the path with the shortest distance in a single AGV manufacturing system is generally the path with the shortest time. However, in a multi-AGV system, multiple AGVs are responsible for different process transportation tasks at the same time, so the path with the shortest distance may be occupied by other AGVs and require a certain waiting time. So, the path with the shortest distance is not necessarily the path with the shortest time. The objective function of the multi-AGV manufacturing system comprises the shortest time path planning to optimize transportation equipment allocation and AGV utilization.

(1) Conflict classification

During the operation of multiple AGVs, there are mainly three types of conflict: node conflict, catch-up conflict, and opposite conflict. Since it is assumed in this paper that AGVs travel at a constant speed during transportation, the catch-up conflicts caused by different speeds will not be discussed in this paper.

 
  1. Opposite conflict

**Figure 2:**
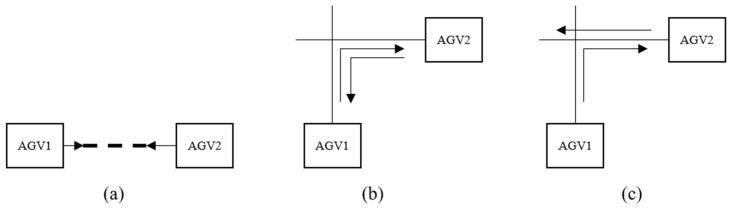
Opposite conflict.

When two AGVs travel towards each other on the same path, only one AGV is allowed to pass on each path at the same time. In this case, two AGVs collide, resulting in a deadlock, as shown in Figure 2a. Similarly, if an AGV moves from one path into the path of another, they will collide when moving in opposite directions, as illustrated in Figure 2b,c.

 
  2. Node conflict

When AGV1 and AGV2 arrive at the same node at the same time, a node conflict will occur at this station, resulting in collision and deadlock.

(2) Regulation principle

 
  1. Shortest path

Under the condition that the speed is fixed, the shorter the path, the shorter the time to execute the transportation task, which can maximize the proportion of the transportation time in the total completion time and ensure that the AGV after the completion of the transportation task can be put into the next scheduling task as soon as possible, saving the transportation resources and the cost generated by the AGV’s own driving.

 
  2. Minimum waiting time

Time is wasted when AGVs collide or choose a suboptimal path.

## 3. Mathematical Models

### 3.1. Integrated Scheduling Problem of Flexible Job-Shop

Based on the above description, the problem under study needs to meet the following assumptions:

(1) Each machine is capable of processing only one job at a time;
(2) Each operation can only be processed on one machine, not split onto two machines;
(3) The AGVs travel at a constant speed in the system, including the time it takes to turn and crossroads;
(4) An AGV is assigned the task of transporting a single job at a time;
(5) It does not consider the charging problem and fault problem of the AGVs, and AGVs are always available;
(6) There is no sequence constraint on the operations between different workpieces;
(7) AGV transportation and machine processing cannot be interrupted;

The meanings of symbols and variables in the established mathematical model are shown in Table 3 [24].

$$\alpha_{ijk}=\{\begin{array}{c} 1,if\;process\;O_{ij}\;is\;processed\;on\;machine\;k\\ 0,else \end{array};$$

$$\beta_{ijpqk}=\{\begin{array}{c} 1,if\;process\;O_{ij}\;is\;before\;process\;O_{pq}\;on\;machine\;k\\ 0,else \end{array};$$

$$x_{ijv}=\{\begin{array}{c} 1,if\;AGV\;v\;is\;responsible\;for\;the\;task\;of\;process\;O_{ij}\;\\ 0,else \end{array};$$

$$y_{ijv}=\{\begin{array}{c} 1,if\;AGV\;v\;is\;responsible\;for\;the\;task\;T_{ijv},\;S^{\prime }T_{ijv}\geq CT_{ijv}\;\\ 0,else \end{array};$$

$$Z_{vst}=\{\begin{array}{c} 1,if\;AGV\;v\;occupies\;node\;s\;at\;time\;t\\ 0,else \end{array};$$

$$A_{s_{1}s_{2}t}\{\begin{array}{c} 1,if\;the\;path\;from\;s_{1}\;to\;the\;adjacent\;node\;s_{2}\;can\;pass\;at\;time\;t,s_{1}\neq s_{2}\\ 0,else \end{array};$$

$$V_{s_{1}s_{2}vt}=\{\begin{array}{c} 1,if\;AGV\;v\;occupies\;the\;path\;from\;s_{1}\;to\;the\;adjacent\;node\;s_{2}\;at\;time\;t,s_{1}\neq s_{2}\\ 0,if\;there\;are\;other\;AGVs\;coming\;in\;the\;opposite\;direction\;on\;the\;same\;path \end{array}$$

In order to ensure that the algorithm meets the corresponding scheduling rules, a set of mathematical models is established as the constraint conditions of the algorithm.

(1) $$C=min\left(C_{max}\right)=min\left(\overset{n}{\underset{i=1}{max}}\left(C_{i}\right)\right)$$

(2) $$w_{opt}=min\left(v\right)\propto min\left(C_{max}\right)$$

(3) $$\overset{m}{\underset{k=1}{\sum }}\alpha_{ijk}=1,\forall i\in \left\{1,2,\ldots ,n\right\},\forall j\in \left\{1,2,\ldots ,u_{i}\right\}$$

(4) $$\begin{array}{c} S_{pqk}+M\left(1-\beta_{ijpqk}\right)\geq C_{ijk},\\ \forall i,p\in \left\{1,2,\ldots ,n\right\},\forall j,q\in \left\{1,2,\ldots ,u_{i}\right\},\forall k\in \left\{1,2,\ldots ,m\right\} \end{array}$$

(5) $$\overset{w}{\underset{v=1}{\sum }}x_{ijv}=1,\forall i\in \left\{1,2,\ldots ,n\right\},\forall j\in \left\{1,2,\ldots ,u_{i}\right\}$$

(6) $$\begin{array}{c} \overset{w}{\underset{v=1}{\sum }}y_{ijv}=1,S^{\prime }T_{i^{\prime }j^{\prime }v}\geq CT_{ijv},\\ \forall i,i^{\prime }\in \left\{1,2,\ldots ,n\right\},\forall j,j^{\prime }\in \left\{1,2,\ldots ,u_{i}\right\},\forall v\in \left\{1,2,\ldots ,w\right\} \end{array}$$

(7) $$\begin{array}{c} C_{ijk}=S_{ijk}+t_{ijk},\\ \forall i\in \left\{1,2,\ldots ,n\right\},\forall j\in \left\{1,2,\ldots ,u_{i}\right\},\forall k\in \left\{1,2,\ldots ,m\right\} \end{array}$$

(8) $$\begin{array}{c} S^{\prime }T_{ijv^{\prime }}\geq CT_{i\left(j-1\right)v}+\overset{m}{\underset{k=1}{\sum }}\alpha_{i\left(j-1\right)k}\cdot t_{i\left(j-1\right)k},\\ \forall i\in \left\{1,2,\ldots ,n\right\},\forall j\in \left\{1,2,\ldots ,u_{i}\right\},\forall v,v^{\prime }\in \left\{1,2,\ldots ,w\right\},\forall k\in \left\{1,2,\ldots ,w\right\} \end{array}$$

(9) $$\begin{array}{c} CT_{ijv}\geq ST_{ijv}+\overset{m}{\underset{k=1}{\sum }}\overset{m}{\underset{k^{\prime }=1}{\sum }}\alpha_{i\left(j-1\right)k^{\prime }}\cdot \alpha_{ijk}\cdot t_{kk^{\prime }},\\ \forall i\in \left\{1,2,\ldots ,n\right\},\forall j\in \left\{1,2,\ldots ,u_{i}\right\},\forall k,k^{\prime }\in \left\{1,2,\ldots ,m\right\},\forall v\in \left\{1,2,\ldots ,w\right\} \end{array}$$

(10) $$\overset{w}{\underset{v=1}{\sum }}z_{vst}\leq 1,\forall s\in \left\{1,2,\ldots ,p\right\},\forall t\in C$$

(11) $$ST_{ijv}\geq max\left(C^{\prime }T_{ijv},C_{i\left(j-1\right)}\right),\forall i\in \left\{1,2,\ldots ,n\right\},\forall j\in \left\{1,2,\ldots ,u_{i}\right\}$$

(12) $$\begin{array}{c} S_{ijk}\geq max\left\{CT_{ijv},max\left\{C_{pqk}|\forall p=1,2,\ldots ,n,q\neq j\right\}\right\},\\ \forall i\in \left\{1,2,\ldots ,n\right\},\forall j\in \left\{1,2,\ldots ,u_{i}\right\},\forall k\in \left\{1,2,\ldots ,m\right\},\forall v\in \left\{1,2,\ldots ,w\right\} \end{array}$$

(13) $$\begin{array}{c} if\;\alpha_{ijk}=1,x_{ijv}=1,C^{\prime }T_{ijv}\geq C_{i\left(j-1\right)k},\\ then\;ST_{ijv}=C^{\prime }T_{ijv};\\ if\;\alpha_{ijk}=1,x_{ijv}=1,C^{\prime }T_{ijv}<C_{i\left(j-1\right)k},\\ then\;ST_{ijv}=C_{i\left(j-1\right)k}.\\ \forall i\in \left\{1,2,\ldots ,n\right\},\forall j\in \left\{1,2,\ldots ,u_{i}\right\},\forall v\in \left\{1,2,\ldots ,w\right\} \end{array}$$

Formula (1) shows that minimizing the maximum makespan is the primary objective. Constraint 2 states that the optimal number of AGVs is equal to the minimum number of AGVs in the job-shop when the maximum completion time is minimum. Constraint 3 indicates that each process can only be processed on one device. Constraint 4 prevents overlapping of the workpiece processing by mandating the start time of a workpiece to come after its completion time. Constraint 5 indicates that each process must be handled by a single AGV for transportation. Constraint 6 ensures that AGVs only move to the next transportation task after completing the previous one to ensure continuity of work and avoid simultaneous tasks. It also ensures that AGVs will not execute two or more transportation tasks at the same time. Constraint 7 indicates that the processing interruptions caused by AGV failure or equipment failure are not considered in the processing process. Constraint 8 indicates that the next no-load transportation task can only commence after the current AGV task is finished. Constraint 9 indicates that the load travel time of the current AGV transportation task should be greater than or equal to the sum of its no-load time and the transportation time required between the two processing machines. This is because the completion time of the last process may be after the time when the AGV arrives at the machine, and the AGV needs to wait for the completion of the last operation before it can execute the transportation task. Constraint 10 indicates that each node can only be occupied by one AGV at a time. Constraint 11 mandates that the AGV cannot start the transportation task until it has completed the preceding operation and arrived at the destination. Constraint 12 indicates that machining can only begin after the end of the load stroke. Constraint 13 restricts the process sequence of the same workpiece and ensures the continuity of the transportation process. If the no-load AGV reaches the position of the processing equipment after the workpiece is processed, the no-load end time is equal to the start time of the load transport. If the no-load AGV arrives before the workpiece is finished, the load transport start time is equal to the workpiece process completion time.

In the production process, the perfect transportation process is similar to this: at the end of the AGV no-load stroke, the last process is just finished; When the AGV load ends, the processing machine just completes the processing task of the current workpiece. If an AGV with no load travel arrives before the completion of the previous process, $C_{i\left(j-1\right)k}>C^{\prime }T_{ijv}$; On the same processing equipment, the completion time of the current workpiece process is greater than the end time of the loading stroke of the next workpiece AGV, $C_{pqk}\cdot \beta_{pqijk}>CT_{ijv}$. Both cases will form invalid waiting times, reduce the utilization rate of AGVs and ultimately affect the final completion time of the whole production.

### 3.2. CFRP

In flexible systems, AGVs need to drive continuously in the workshop to transport the workpieces and materials to their destination and need to address and change the path constantly. Therefore, it is necessary to solve various conflicts that may be encountered in path planning at the algorithm level [25]. Traditional path-planning strategies are usually divided into two types. One path strategy prioritizes the optimal path and chooses to wait in place or in other idle paths according to the conflict type to ensure the shortest AGV distance. Another routing strategy does not consider path conflicts but only ensures the principle of avoiding conflicts during AGV movement. Once a path or node is occupied, the second-best feasible path is selected. Neither of these two strategies can guarantee the shortest transportation time for AGVs. On the basis of these two strategies, a new recursive discriminant strategy is proposed in this paper, which not only solves the path conflict problem but also plans an accurate conflict-free path for AGVs to ensure the shortest transportation time of AGVs.

In the multi-AGV manufacturing system, the current problem to be solved is how to make the car find a sub-optimal path to reach the destination with the shortest time and minimum cost loss while avoiding a collision. We provided each car with a different task, entered the shop in turn, completed the task, and arrived at the destination. The map adopts a single-row bidirectional job-shop layout, so before establishing a mathematical model for the planning problem, basic assumptions should be made about the system where the AGV resides [26]:

(1) One AGV can only perform one task at a time.
(2) All AGVs move at a constant velocity and possess identical features.
(3) Breakdowns and charging problems are not taken into account.
(4) Once AGV is working, it is not allowed to be interrupted.

The mathematical model employs the following set of symbols and variables:

w: index of AGVs

$$k_{v}:\;\mathrm{set}\;\mathrm{of}\;\mathrm{AGVs},\;v\in \left\{1,2,\ldots ,w\right\};$$

$$N_{s}:\;\mathrm{set}\;\mathrm{of}\;\mathrm{nodes},\;s\in \left\{1,2,\ldots ,p\right\},\;p\;\mathrm{represents}\;\mathrm{the}\;\mathrm{node}\;\mathrm{number};$$

$$t_{{ss}^{\prime }}:\;\mathrm{the}\;\mathrm{travel}\;\mathrm{time}\;\mathrm{of}\;\mathrm{the}\;\mathrm{AGV}\;\mathrm{from}\;\mathrm{notes}\;s\;\mathrm{to}\;\mathrm{node}\;s^{\prime },\;s,\;s^{\prime }\in \left\{1,2,\ldots ,p\right\},\;s\;\neq \;s^{\prime };$$

$$Z_{vst}=\{\begin{array}{c} 1,if\;AGV\;v\;occupies\;node\;s\;at\;time\;t\\ 0,else \end{array};$$

$$A_{s_{1}s_{2}t}\{\begin{array}{c} 1,if\;the\;path\;from\;s_{1}\;to\;the\;adjacent\;node\;s_{2}\;can\;pass\;at\;time\;t,s_{1}\neq s_{2}\\ 0,else \end{array};$$

$$V_{s_{1}s_{2}vt}=\{\begin{array}{c} 1,if\;AGV\;v\;occupies\;the\;path\;from\;s_{1}\;to\;the\;adjacent\;node\;s_{2}\;at\;time\;t,s_{1}\neq s_{2}\\ 0,if\;there\;are\;other\;AGVs\;coming\;in\;the\;opposite\;direction\;on\;the\;same\;path \end{array}$$

AGVs travel at a constant speed, and the mathematical model takes the shortest total travel time of all AGVs as the objective function, as shown in Formula (14).

(14) $$minZ=min\overset{w}{\underset{v=1}{\sum }}Q_{v}$$

**Figure 3:**
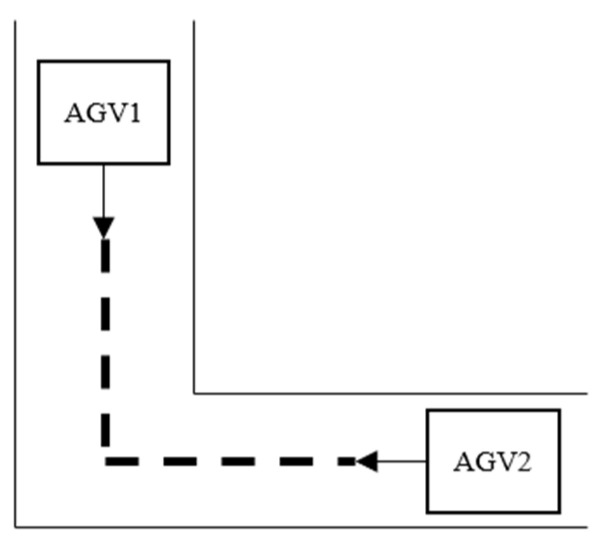
Node conflict.

Firstly, mathematical modeling is carried out for possible path conflicts in path planning. Node conflict is caused by more than one car arriving at a node at the same time. Formula (15) indicates that only one car can occupy a node at the same time, as shown in Figure 3.

(15) $$\overset{w}{\underset{v=1}{\sum }}z_{vst}\leq 1,\forall s\in \left\{1,2,\ldots ,p\right\}$$

If two AGVs do not arrive at a node at the same time, but the AGV closest to the node needs to drive to another occupied path through this node, and the AGV near the node is on the opposite path, the AGV near the node has two choices: (1) Wait for another car to release the path; (2) Replace another suboptimal path. For the first case, suppose there are two AGVs; AGV2 is closer to the node $s_{2}$, AGV1 turns to the adjacent path of AGV2, as shown in Figure 2b, then the mathematical model of such conflicts is as follows:

(16) $$\begin{array}{c} If\;V_{s_{1}s_{2}v_{1}t}*V_{s_{3}s_{2}v_{2}t}=1,\\ s1,s2,s3\in \left\{1,2,\ldots ,p\right\},\;s1\neq s2\neq s3,v1,v2\in \left\{1,2,\ldots ,w\right\},v1\neq v2,\\ Then\;A_{s_{2}s_{3}\left(t+t_{s_{3}s_{2}}\right)}=1 \end{array}$$

For the conflict type in Figure 2b, if AGV1 is closer to the node $s_{2}$ and turns to the adjacent path of AGV2; AGV1 has two choices: wait or change paths. Assuming that the time used by the original optimal path to reach the end node is t, the total waiting time of the car at the intersection is Δt, and the time required by the replacement of the suboptimal path is $t^{\prime }$. However, since the suboptimal path also needs to consider the path conflict problem, this path optimization strategy is easy to fall into the local optimal. In order to reduce the solution space, the suboptimal solution that does not consider path conflicts is first determined, and the suboptimal solution $t+\Delta t\geq \min\left[t^{\prime },t^{\prime \prime },t^{\prime \prime \prime }\ldots t^{n}\right]$ that is less than the total transportation time of the optimal path selected. Then, considering the path conflict of the second-best path, the car usually has multiple second-best paths from one device to another device. A recursive strategy is used to plan a global shortest-time path judgment method for AGV, as shown in Formula (17).

Assuming that the shortest path of AGV from node p to node $p^{\prime }$ needs to pass through n nodes, which will be encountered in the driving process, then the AGV will encounter at most n+1 path conflicts of the same type when it reaches node $p^{\prime }$, and it needs to determine whether to choose the second-best path each time. Therefore $\sum \Delta t={\Delta \Delta }_{1}+\Delta t_{2}+\Delta t_{3}\ldots +\Delta_{n+1}$, the waiting time for the conflict of the second-best path is the same. According to the comparison of the transportation time and waiting time of the sub-optimal path, the optimal path scheme is selected. If Formula (17) holds, the optimal path is selected; otherwise, the suboptimal path is selected.

(17) $$t+\sum \Delta t\leq min\left[t^{\prime }+\sum \Delta t^{\prime },t^{\prime \prime }+\sum \Delta t^{\prime \prime },t^{\prime \prime \prime }+\sum \Delta t^{\prime \prime \prime }\ldots \right]$$

For the type of locking in Figure 2c, the general adjustment strategy is to let the car that first arrives at the conflict node change the path and, at the same time, ensure that there is a second optimal path available for replacement. Assuming that the AGV1 is closer to the conflict node $s_{2}$, the adjustment strategy is shown in Formula (18).

(18) $$\begin{array}{c} \begin{array}{c} IfV_{s_{1}s_{2}v_{1}t}\;*\;V_{s_{3}s_{2}v_{2}t}=1,A_{s_{2}s_{1}\left(t+t_{s_{1}s_{2}}\right)}=1,A_{s_{2}s_{3}\left(t+t_{s_{1}s_{2}}\right)}=0,\#\\ s_{1},s_{2},s_{3},s_{4},s_{5}\in \left\{1,2,\ldots ,p\right\},s_{1}\neq s_{2}\neq s_{3}\neq s_{4}\neq s_{5},s_{2}\;is\;an\;adjacent\;node\;to\;s_{1},s_{3},s_{4},s_{5},\\ v_{1},v_{2}\in \left\{1,2,\ldots ,w\right\},v_{1}\neq v_{2},\\ Then\;A_{s_{2}s_{4}\left(t+t_{s_{1}s_{2}}\right)}+A_{s_{2}s_{5}\left(t+t_{s_{1}s_{2}}\right)}\geq 1 \end{array} \end{array}$$

$V_{s_{1}s_{2}v_{1}t}*V_{s_{3}s_{2}v_{2}t}=1$ indicates that the paths from $s_{1}$ to $s_{2}$ and from $s_{3}$ to $s_{2}$ are occupied by AGV1 and AGV2 respectively. $A_{s_{2}s_{1}\left(t+t_{s_{1}s_{2}}\right)}=1$ indicates that after time $t_{s_{1}s_{2}}$, AGV1 first reaches the conflict node $s_{2}$, but AGV2 still occupies the path from $s_{3}$ to $s_{2}$. $A_{s_{2}s_{4}\left(t+t_{s_{1}s_{2}}\right)}+A_{s_{2}s_{5}\left(t+t_{s_{1}s_{2}}\right)}\geq 1$ means that at least one of the other two paths from $s_{2}$ is a viable path.

If the conflict node $s_{2}$ in Figure 2b has only three adjacent nodes, which means deadlock occurs at the T-junction, then the adjustment strategy is shown as follows.

(19) $$\begin{array}{c} If\;V_{s_{1}s_{2}v_{1}t}\;*\;V_{s_{3}s_{2}v_{2}t}=1,A_{s_{2}s_{1}\left(t+t_{s_{1}s_{2}}\right)}=1,A_{s_{2}s_{3}\left(t+t_{s_{1}s_{2}}\right)}=0\\ s_{1},s_{2},s_{3},s_{4}\in \left\{1,2,\ldots ,p\right\},s_{1}\neq s_{2}\neq s_{3}\neq s_{4},s_{2}\;is\;an\;adjacent\;node\;to\;s_{1},s_{3},s_{4},\\ v_{1},v_{2}\in \left\{1,2,\ldots ,w\right\},v_{1}\neq v_{2},\\ Then\;A_{s_{2}s_{4}\left(t+t_{s_{1}s_{2}}\right)}\geq 1 \end{array}$$

Formulas (18) and (19) are designed to ensure that there is room for adjustment of such conflicts. If the two formulas are valid, the earliest AGV arriving at the node can choose to wait on the free path until the original path is released or choose the second-best path. For specific adjustment schemes, refer to Formula (17). In cases when Formulas (18) and (19) are not valid, Figure 2b is categorized under the same type of unadjustable deadlock as Figure 2a.

The situation description and adjustment scheme in Figure 2a: If a path is occupied by an AGV, then this path does not allow AGVs in the opposite direction to enter at the same time. Since the AGVs in the system travel at the same speed, two AGVs traveling in the same direction are allowed on the same path. Formula (20) ensures that multiple AGVs can travel in the same direction on the same path and that two or more AGVs are not allowed to travel in the same direction on the same path.

(20) $$\begin{array}{c} If\;V_{s_{1}s_{2}v_{1}t}=1,\\ s_{1},s_{2}\in \left\{1,2,\ldots ,p\right\},\;s_{1}\neq s_{2},\\ v_{1},v_{2},v_{3}\in \left\{1,2,\ldots ,w\right\},v_{1}\neq v_{2}\neq v_{3},\\ Then\;V_{s_{1}s_{2}v_{2}t}=1,\;V_{s_{2}s_{1}v_{3}t}=0 \end{array}$$

## 4. Approach Statement

Integrated scheduling of flexible job-shop is solved in two stages: production scheduling and AGV scheduling. In the first stage, a hybrid genetic algorithm is used to solve the production scheduling problem. In the second stage, according to the production scheduling scheme obtained in the first stage, an AGV coding-based genetic algorithm is used to study the AGV scheduling problem. At the same time, AGV routing problems and path conflicts are taken into account, and finally, a better feasible scheduling scheme is obtained.

### 4.1. Production Scheduling

#### 4.1.1. Encoding Method

Genetic algorithms are largely determined by the choice of an appropriate encoding scheme. It is desirable to keep the chromosome length as short as possible to reduce unnecessary computational burden and focus on promising solution alternatives. For the exploration of all potential solutions within the search space, the encoding method should be capable of accurately representing them. In the case of scheduling operations, there are parallels between the job-shop scheduling problem and the first part of the studied problem in theory. Thus, an operation-based encoding method is used in the approach.

**Figure 4:**
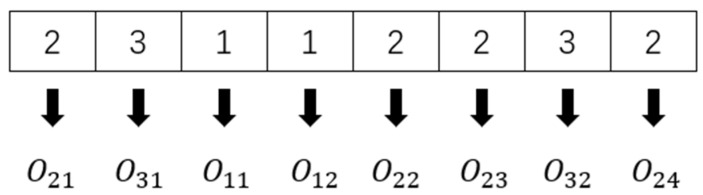
Operations-based encoding.

Figure 4 depicts the encoding method. The sum of all operations represents the length of the chromosome. Assume that the operations chromosome is [2 3 1 1 2 2 3 2]; the first 2 represents the first operation $O_{21}$ of workpiece $J_{2}$, and the second 2 represents the second process $O_{22}$ of workpiece *J*_2_. The rest can be performed using the same method. So, the sequence of the operations is $O_{21}$, $O_{31}$, $O_{11}$, $O_{12}$, $O_{22}$, $O_{23}$, $O_{32}$, $O_{24}$.

#### 4.1.2. Decoding Method

The proposed approach utilizes the Vehicle Assignment Algorithm (VAA) to address the vehicle assignment and scheduling issues integrated into the decoding method. The pseudocode for the decoding method is shown in Algorithm 1.
**Algorithm 1**: Decoding method  **for** each operation op in the chromosome **do**machine mc = processing machine of oppredecessor pre = preceding operation of op**if** pre == NULL **then**start_time st = 0**else**start_time st = pre. completion_time**end if**number_of_scheduled_ops ns = number of scheduled operations on machine mc**if** ns > 0 **then**last_scheduled_op sched = last scheduled operation on machine mccompletion_time ct = sched. completion_time**if** st < ct **then**start_time st = ct**end if****end if**completion_time ct = st + op. processing_timeschedule operation op on machine mc with completion time ct**end for** 

During the decoding process, the VAA acquires crucial information, such as the current operation (op), its recommended start time (st), and the preceding operation within the sequence (pre). The VAA determines which AGV can complete the transportation in the least amount of time and adjusts the start time st to account for transportation duration. The pseudocode for the VAA is demonstrated in Algorithm 2.
**Algorithm 2**: Vehicle assignment algorithm  min_time_to_finish = infinitybest_AGV = null**for** each AGV in available_AGVs **do**S = AGV. last_trip_destination**if** pre != null **then**D = processing machine responsible for operation pre**else**D = station for loading or unloading materials**end if**dht = travel time between S and Dtime_to_finish = max(pre.completion_time, AGV.last_trip_finish_time) + dht**if** time_to_finish < min_time_to_finish **then**min_time_to_finish = time_to_finishbest_AGV = AGV**end if****end for**add_trips_to_best_AGV (best_AGV, operation_op)time_to_start = best_AGV. last_trip_finish_time**if** st < time_to_start **then**st = time_to_start**end if**

The VAA, an optimization algorithm, is designed to determine the AGV that provides the earliest possible start time for a given operation processed by its allocated machine. The VAA uses the maximum value between the AGV’s last trip finish time and the preceding operation completion time in evaluating the start time for a deadheading trip, considering the required deadheading trip time. The calculation of start time depends on adding the available start times for deadheading trips, which are exclusively related to the deadheading trip durations of AGVs due to their identical loaded trip times.

The decoding method employed in this algorithm is based on a critical observation that a machine schedule generated using the operations-based coding approach tends to stretch when vehicle transportation times are factored. This is due to a shift in each operation’s start time. During operation, the VAA aims to minimize these changes in start times to reduce the makespan. In doing so, the algorithm searches for AGVs that have minimum completion times for each trip, thus minimizing the shift in start time for each operation.

#### 4.1.3. Crossover Operator

Uniform crossover is a crossover method utilized in generating a new chromosome from two matching chromosomes. In this method, each gene in the chromosome is exchanged with a predetermined probability. Uniform crossover can be seen as a variation of the multi-point crossover method, with specific rules in place to ensure the preservation of the parent genes and maintain the coding structure.

**Figure 5:**
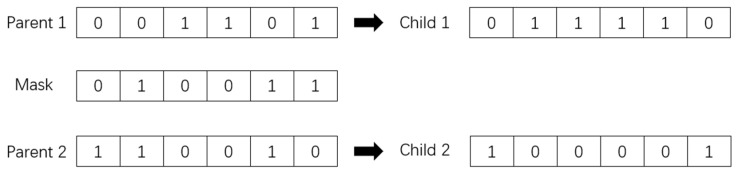
Uniform crossover.

To generate a new chromosome via a uniform crossover, a crossover mask is randomly generated with the same length as the parent chromosomes. The mask is then applied to the two matched chromosomes. Genes corresponding to “1” in the mask are swapped between chromosomes, while genes that correspond to “0” remain unchanged. By randomly crossing every bit of the parent chromosomes with the same probability, two child chromosomes are eventually created. Figure 5 provides a visual representation of the uniform crossover mechanism.

#### 4.1.4. Mutation Operator

**Figure 6:**
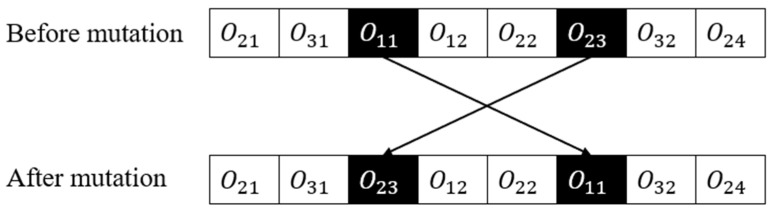
Swap mutation.

Mutation operators refer to the genetic algorithm components that are responsible for introducing genetic diversity into the population of potential solutions. These operators randomly modify one or more genes or components of a candidate solution, essentially creating a new candidate solution that is slightly different from its parent. This diversity helps the algorithm to move beyond local optima and discover better solutions in unexplored areas of the search space. In swap mutation, two genes are randomly selected from the parent chromosome, and then their position is swapped, as illustrated in Figure 6.

#### 4.1.5. The Schedule Recovery Algorithm

The schedule recovery algorithm is a crucial step after applying the genetic operators of crossover and mutation in order to ensure that technological restrictions are met during the GA search. This algorithm is accomplished by rearranging the operations while ensuring that each operation in the sequence is executed within the specified technological constraints. Ultimately, the algorithm aims to produce a feasible and optimized schedule that meets all the necessary constraints.

**Figure 7:**
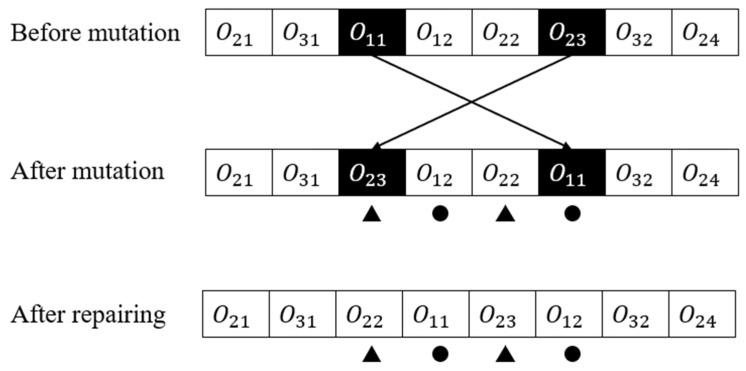
The schedule recovery algorithm.

As shown in Figure 7, the operation $o_{22}$ is behind operation $o_{23}$ and operation $o_{11}$ is behind operation $o_{12}$ after swap mutation. In this case, the schedule recovery algorithm is needed. The pseudocode for the schedule recovery algorithm is demonstrated in Algorithm 3.
**Algorithm 3**: The schedule recovery algorithm   **for** each operation op **in** the chromosome **do**opi = operation located at position iprei = preceding operation of opi**if** prei≠NULL **then****for** each operation op **in** the chromosome **do**opj← operation located at position j**if** prei=opj **then**Swap positions of opi and opj**end if****end for****end if****end for**

#### 4.1.6. Elite Preservation

The paper employs an elite preservation strategy to minimize the loss of the best-fit individuals during the GA run. This strategy entails preserving a group of the best-fit individuals across the generations and updating them with better individuals when they emerge. The size of the set remains constant throughout the run since it is specified by the user during the algorithm’s configuration.

### 4.2. AGV Scheduling

Product scheduling result is a known condition that has been solved in the first stage. Therefore, in the second stage, the scheduling scheme of the processing machine, the processing time of the machine, and the process sequence on each processing machine are taken as the input conditions. Then the problems to be solved in the second stage become the AGV routing problem and the scheduling problem of AGVs. In the second stage, the scheduling scheme of the processing machine is decomposed into corresponding transportation tasks, and the scheduling problem of AGVs becomes the process of assigning different transportation tasks to AGVs while considering the problem of path conflict. AGVs are equipped with sensors for the purpose of providing real-time location data. Formulating conflict-free paths ensures that AGVs will not collide accidentally.

#### 4.2.1. Dijkstra

The Dijkstra algorithm is a classical path planning algorithm based on graph theory G=(V,E) to solve the shortest distance between two nodes. Where V represents the set of all nodes in the topological map, and E represents the set of all lines in the topological map. According to the layout of the job-shop, the important equipment position is set as the node, the path that can be directly connected between the node and the node is represented by the line, and the corresponding edge is assigned the corresponding weight according to the distance of the connecting path or through the time. In this study, unit time is used to represent the weight between the two nodes so that the complex shop environment can be abstracted into the topological map represented by the node and the line. Topological maps are expressed in the form of an Adjacency Matrix (AM) in the algorithm. The core of the Dijkstra algorithm is to take the start node as the center and find the optimal path with the smallest sum of ownership values to the end node according to the weights of the adjacency matrix and lines between nodes. The procedures are as follows:

Step 1: Set initial conditions: start node $S_{0}$ and end node $U_{0}$. Calculate the shortest distance from the start node to the end node.

Step 2: Set and initialize the node set S (the set of nodes with the lowest weight that has been calculated so far) and U (the weight of $S_{0}$ to the remaining nodes). Where S contains $S_{0}$, and the distance from $S_{0}$ to $S_{0}$ is known to be 0; U contains other nodes except $S_{0}$. The distance between nodes is initialized. If two nodes are adjacent, the weight is the weight of the line between two nodes; if not, the weight is infinite.

Step 3: Go through all nodes in the set U, determine the adjacency $S_{k}$ closest to $S_{0}$, delete $S_{k}$ from the set U and add $S_{k}$ to the set S, and determine whether $S_{k}$ is $U_{0}$. If yes, the algorithm terminates and $S_{k}$ is the shortest path in the map; otherwise, update the weight $G_{0\to k}$ of $S_{0}$ to $S_{k}$ and jump to the next step.

Step 4: Calculate $S_{k}$ as the start node to traverse all nodes in set U and take the node $S_{k+1}$ with the shortest distance from $S_{k}$, delete $S_{k+1}$ from set U and add it to set S, and update the weight of $S_{0}$ in set U to the other nodes. If $G_{0\to k+1}<G_{0\to k}+G_{k\to k+1}$, update the weight of $G_{0\to k+1}$ in set S; Otherwise, proceed to the next step.

Step 5: Determine whether $S_{k+1}$ is $U_{0}$. If it is, terminate the algorithm to obtain the shortest path information and minimum weight. Otherwise, loop steps 4 and 5 until you find the end node $U_{0}$.

#### 4.2.2. Time Window

The basic idea of a time window is to mark the direction of the occupied path. Other AGVs cannot pass through this path in the opposite or the same direction. AGVs can only wait or change other paths until the occupied path ends and other AGVs can pass through. In the time window method, path L is often divided into two parts: idle time window set $F_{L}$ and reserved time window set $R_{L}$. The commonly used mathematical model of the time window is as follows:

(21) $$F_{L}=\left\{f^{k}=\left[a^{k},b^{k}\right]\right\}$$

(22) $$R_{L}=\left\{r^{k}=\left[c^{k},d^{k}\right]\right\}$$

In Formula (21), $f^{k}$ represents the idle time period of path L, and k represents the $k_{th}$ idle time period, starting from $a^{k}$ and ending from $b^{k}$. Similarly, $r^{k}$ in Formula (22) represents the occupied period of path L, starting from $c^{k}$ and ending with $d^{k}$.

#### 4.2.3. Algorithm Design

Since the scheduling problem of the processing machine has been solved, based on the mathematical model of the integrated scheduling problem, the mathematical scheduling model of the second stage only needs to consider the processing sequence constraint of the same workpiece, the process sequence constraint of the workpiece on the same equipment and the AGV scheduling problem.

The solution of the AGV routing problem and path conflict problem refers to the solution strategy in Section 3.2. Taking the minimum completion time of the maximum transportation process as the objective function, the mathematical model of the second stage is as follows:

(23) $$C=min\left(CT_{max}\right)=min\left(\overset{n}{\underset{i=1}{max}}\left(CT_{iu_{i}\nu }\right)\right)$$

(24) $$\begin{array}{c} if\;\alpha_{ijk}=\alpha_{pqk}=1,\beta_{ijpqk}=1,\\ C_{ijk}+ITW\leq S_{pqk},\\ \forall i,p\in \left\{1,2,\ldots ,n\right\},\forall j,q\in \left\{1,2,\ldots ,u_{i}\right\},k\in \left\{1,2,\ldots ,m\right\} \end{array}$$

Formula (23) takes the minimum completion time of the maximum transportation process as the objective function. Formula (24) guarantees that workpieces are processed in a specific sequence on each processing machine.

A genetic algorithm that employs AGV-encoding is utilized in the second stage to resolve AGV scheduling and conflict-free routing issues, with the incorporation of the time window-based Dijkstra algorithm.

1. Encoding method

**Figure 8:**
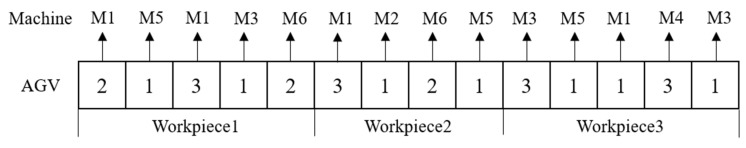
Encoding and decoding method based on AGV number and obtained scheduling scheme.

In this section, AGV-based encoding is adopted. Since the working procedure of the workpiece on each machine is known, chromosomes are arranged according to the sequence of workpieces and their operations. The number on the chromosome represents the AGV number responsible for transporting that operation. For example, there are five genes in the coding position of Workpiece 1, which respectively imply five operations of Workpiece 1. The first operation of Workpiece 1 will be transported to machine M1 by AGV2, as shown in Figure 8.

When the objective function is to minimize the maximum completion time, the maximum number of AGVs usually has an upper limit. In order to rationally allocate logistics equipment resources, the upper limit of the number of AGVs in the chromosome is set, and the variation of the mutation operator [1,…w]  is set. The number of AGVs is cross-mutated. Calculate the fitness function of each iteration result, and the number of AGVs is finally obtained when the maximum completion time is minimum. Finally, output the optimal scheduling scheme.

2. Decoding method

AGVs are assigned to transport tasks according to the number of AGVs in chromosomes. The AGV makes route planning according to the assigned transportation tasks and finds the shortest conflict-free path so as to transport the last workpiece to the finished product warehouse as the maximum completion time. Specific decoding steps are shown as follows:

Step 1: Transform the chromosome based on the process code of the gene chain into a specific process chain.

Step 2: Read every gene of the process chain according to the sequence of different workpiece areas. The gene number indicates that the corresponding process is responsible for $O_{ij}$. Read the implied information of the gene, the corresponding processing machine k, the processing time of the corresponding machine is $t_{ijk}$, and the processing sequence of the process on the machine k.

Step 3: Make sure $\alpha_{\mathrm{ijk}}=\alpha_{\mathrm{pqk}}=1,\beta_{ijpqk}=1$. According to the completion time $C_{i\left(j-1\right)k^{\prime }}$ of the tight preceding procedure $O_{i\left(j-1\right)}$ of procedure $O_{ij}$, the completion time $C_{i^{\prime }j^{\prime }k}$ of the tight preceding procedure in machine k, the number of the AGV in charge of the processing task and the location of the node where the AGV is located, determine the time $S^{\prime }T_{ijv}$ when the AGV can start no-load transportation.

Step 4: Route planning for no-load transportation. According to the available time $S^{\prime }T_{ijv}$ of the AGV in charge of the transportation task (or the end time $CT_{i^{\prime }j^{\prime }v}$ of load transportation of the last process that v is responsible for), the location node, the node position S of the machine in charge of the process $O_{i\left(j-1\right)}$ and the adjacency matrix $A_{dj}M$, the next node N of the shortest path of no-load transportation, is determined by the Dijkstra algorithm. Go to Step 7.

Step 5: Route planning for load transportation. Compare the completion time of the tight preceding process $C_{i\left(j-1\right)k^{\prime }}$, if $C_{i\left(j-1\right)k^{\prime }}>C^{\prime }T_{ijv}$, then $ST_{ijv}=C_{i\left(j-1\right)k^{\prime }}$ (The transportation of a load begins only after completion of the preceding process directly connected to it). On the contrary, if the AGV arrives at k when the tight preceding process has been completed, the completion time of the tight preceding process is regarded as the start time of load transportation, that is, $ST_{ijv}=C^{\prime }T_{ijv}$.

Step 6: According to the adjacency matrix $A_{dj}M$, the position node S of the tight preceding operation, the position node T of the processing machine responsible for the current operation, and the start time of the load transportation use the Dijkstra algorithm based on the time window to calculate the next node N of the load transportation path. Go to Step 7 and repeat Step 5 until N=T.

Step 7: Path conflict detection. Determine if the planned paths conflict with each other based on their respective time windows, and if there are no conflicts identified, proceed to Step 8. If there are conflicts, proceed to Step 9.

Step 8: Update the time window of each section on the topology map. In the no-load transport phase, check whether N=S. If yes, go back to Step 5. Otherwise, return to step 4 until N=S is true, and then return to step 5. If N=T during the load transport phase, generate the shortest path plan and revise the time window of each section accordingly. Go to Step 11. Otherwise, continue to search for the next node of the shortest path through the Dijkstra algorithm and return to Step 7.

Step 9: Detect the path conflict type.

Step 10: Determine if the transportation is over. If N=S, return to Step 5, output the shortest path, and update the time windows of each section; If N=T, compare the completion time of the previous process $C_{i^{\prime }j^{\prime }k}$ of k. If $C_{i^{\prime }j^{\prime }k}>CT_{ijv}$, the start time of $O_{ij\;}S_{ijk}=C_{i^{\prime }j^{\prime }k}$; if $C_{i^{\prime }j^{\prime }k}<CT_{ijv}$, the start time is equal to the end time of load transportation, that is $S_{ijk}=CT_{ijv}$. Then, output the shortest path and update the time windows of each section. Go to Step 11. Otherwise, select the suboptimal path and go to Step 7.

Step 11: Output the start time, end time, and available time based on the shortest path and time window of the process.

Step 12: Iterate Steps 3–11 until the selection of all workpieces and process transportation tasks has been accomplished, and output the path planning scheme, that is, the final scheduling scheme, according to the time set of each workpiece (such as the time and sequence of each working process).

## 5. Computational Results

### 5.1. Parameter Settings

The performance of the algorithm is heavily affected by the crossover probability $P_{c}$ and mutation probability $P_{m}$, which are critical components of the algorithm. An increase in the crossover probability improves the ease of generating new individuals. However, setting the crossover probability too high might cause the individuals’ structures to disintegrate. Meanwhile, decreasing the crossover probability might significantly reduce the algorithm’s search efficiency. When the mutation probability value is too low, producing new individuals becomes challenging. Conversely, an extremely high mutation probability can convert the genetic algorithm into a random search algorithm, significantly reducing the algorithm’s efficiency [27].

**Figure 9:**
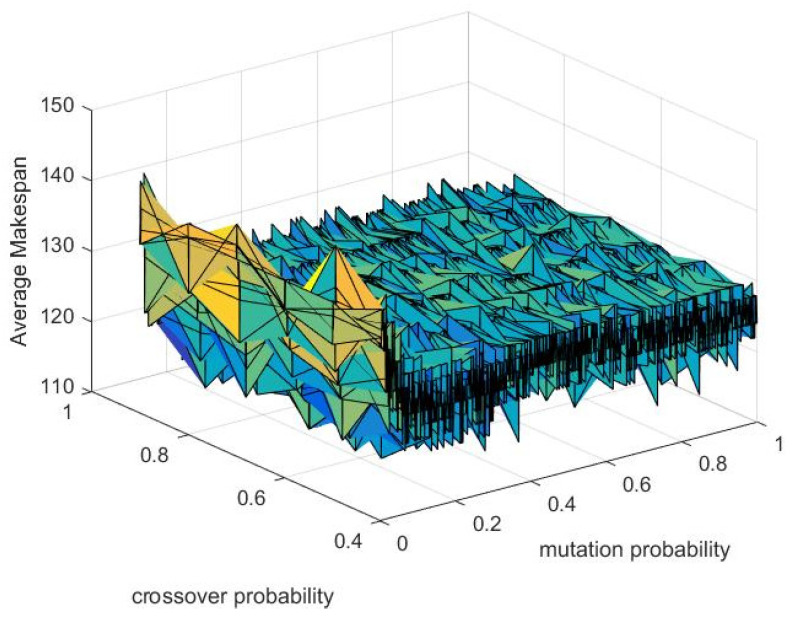
The performance of GA under different probabilities of crossover and mutation.

Experiments are conducted on a Windows 10 computer with an Intel Core i7-8750H CPU, clocked at 2.50 GHz, and 16.00 GB of RAM, using Python programming language version 3.9. The performance of the genetic algorithm is analyzed by manipulating the crossover and mutation probabilities, ranging from 0.4 to 1.0 and 0.0001 to 1.0, respectively [28]. Based on 20 independent genetic algorithm trials, the algorithm’s effectiveness is assessed by averaging the makespan. The results of the study concluded that higher probabilities of crossover and mutation result in more optimal solutions. As shown in Figure 9, the optimal range of crossover probability is determined to be between 0.8 and 1.0, while the ideal range for mutation probability is confirmed at a range between 0.4 to 1.0. In addition, the algorithm’s uniform performance within these parametric ranges is presented.

### 5.2. Comparative Experiment

The comparative experiment utilizes the collection of 82 test problems suggested by Ulusoy and Bilge. These problems consist of four workshop layouts and 10 production tasks and are divided into two groups according to the proportion of transportation time and processing time. For 40 cases with t/p>0.25 and 42 cases with t/p<0.25, t represents the transportation time between different processing machines, and p represents the time of operation. When t/p>0.25, AGV transportation time takes up a large proportion. The purpose is to verify the algorithm’s searching ability for integrated scheduling and path planning. When t/p<0.25, problem number mantissa 0 means that all processing time is doubled, and mantissa 1 means that all processing time is multiplied by three. At the same time, the path in the map is halved to reduce the influence factors of path planning in the problem. In order to ensure the consistency of data comparison, this paper also adopts the same path planning strategy, AGV number, and process constraints. Experiments use the same map as the classical example.

The algorithm settings used in this study entail a crossover probability of 0.9, a mutation probability of 0.5, a population size of 100, an elite set size of 10, number of generations set to 100, with a uniform crossover as the crossover operator, swap mutation as the mutation operator, roulette wheel as the selection scheme, and 20 genetic algorithm (GA) runs. Results are shown in the proposed algorithm in Table 4 and Table 5. The LB value is the theoretical optimal value obtained by the lower limit method proposed by Ulusoy. The STW value is the optimal value obtained by the sliding time window heuristic proposed by Bilge. The UGA value is the optimal value obtained by the genetic algorithm proposed by Ulusoy. The improvement value is defined as the discrepancy between the optimal outcomes achieved by the UGA and those achieved by the proposed algorithm.

In general, the proposed algorithm is in good agreement with the LB value obtained by the lower limit method proposed by Ulusoy. In comparison with the STW heuristic, the proposed algorithm outperforms it in all problems examined, while the UGA underperforms in six cases. Moreover, the proposed algorithm delivers better solutions for twenty-one problems having t/p ratios exceeding 0.25, surpassing the UGA’s improved solutions in only four. However, for the problems with t/p ratios below 0.25, the UGA outperforms the proposed GA in six issues, while the proposed GA can only achieve for three issues.

Abdelmaguid [29] has invalidated the UGA’s results for seven problems, namely EX81, EX82, EX83, EX810, EX820, EX830, and EX840, where the STW used the best methods. If the results generated by the UGA are ignored, it becomes evident that the proposed algorithm outperforms the UGA by a significant margin. Specifically, the proposed algorithm obtains improved solutions for twenty-four problems, while the UGA is only better in three problems.

### 5.3. Case Verification

**Figure 10:**
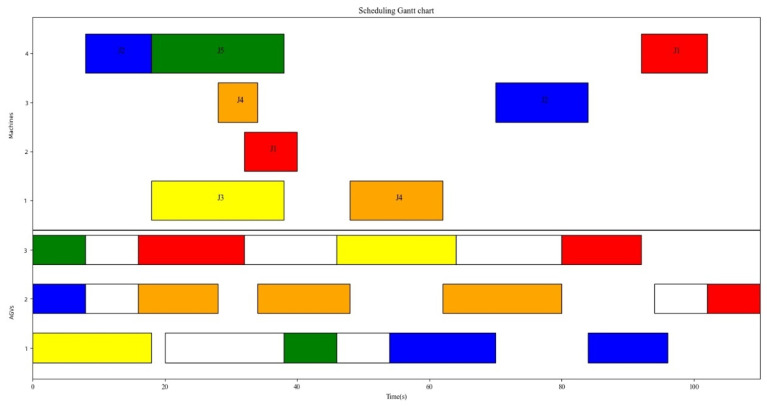
Gantt chart of production scheduling.

We solve the case presented in Section 2.1 to verify both the efficacy and feasibility of the proposed algorithm with regard to addressing path conflicts. The white rectangular in Figure 10 represents the AGVs’ deadheading trips. The various colors correspond to different workpieces: red represents J1, blue represents J2, yellow represents J3, orange represents J4, and green represents J5. Figure 10 illustrates that the makespan for production scheduling is 110 s.

**Figure 11:**
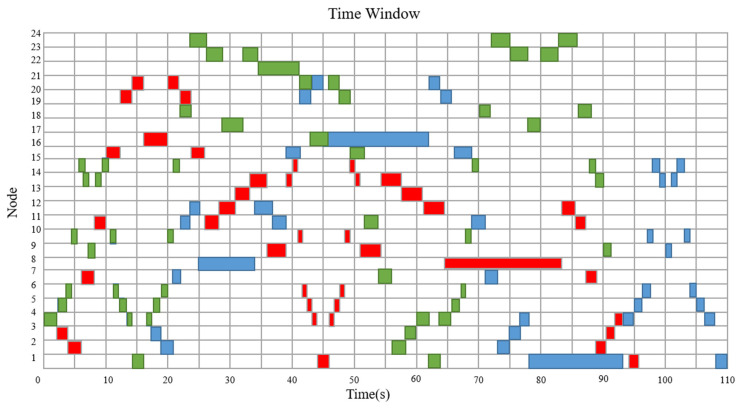
Time window without considering path conflicts.

**Figure 12:**
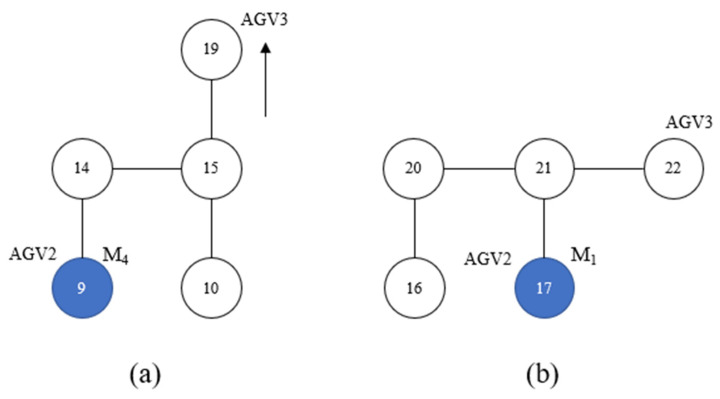
Path conflicts happen in production scheduling.

Figure 11 illustrates the time window without considering path conflicts, where the red color represents AGV1, blue color represents AGV2, and green color represents AGV3. AGV1, AGV2, and AGV3 depart from node 4 at the same time, so we have to let them pass in order of priority. As is shown in Figure 12a, the route of AGV2 from 7 s to 14 s is “10-15-14-9-14-15-10”, which is the same as that of AGV3, so AGV3 cannot pass. According to the scheme, AGV3 will go to node 19 from node 15 and wait until AGV2 leaves node 15. Figure 12b illustrates another conflict at 47 s. AGV2 and AGV3 need to go to M1, so AGV3 will have to stay at node 22 until AGV2 leaves node 21.

**Figure 13:**
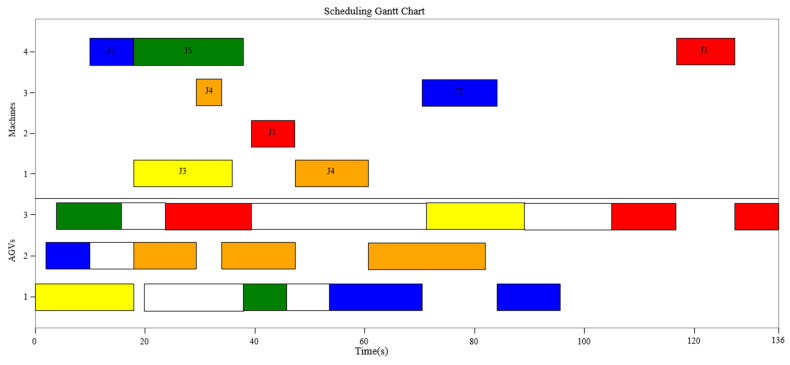
Gantt chart of final scheduling.

**Figure 14:**
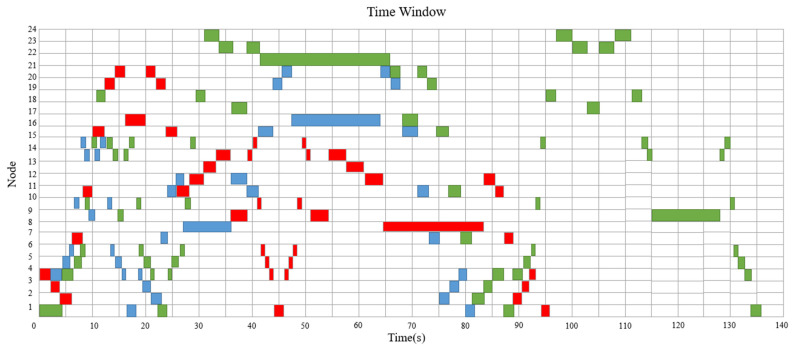
Time window of final scheduling.

Figure 13 and Figure 14 show the Gantt Chart and the time window of final scheduling after optimizing the path conflicts. The route of AGV1 is “L/U-M1-M4-L/U-M4-M3-L/U”. The route of AGV2 is “L/U-M4-L/U-M3-M1-L/U”. The route of AGV3 is “L/U-M4-L/U-M2-M1-L/U-M2-M4-L/U”. All path conflicts among AGVs are eliminated. Finally, the maximum completion time is determined to be 136 s.

## 6. Conclusions

This study addresses the integrated scheduling problem of flexible job-shops that considers CFRP, with the objective of minimizing the maximum makespan. The study develops a hybrid genetic algorithm for production scheduling, with the optimal range of crossover and mutation probabilities identified as 0.8–1.0 and 0.4–1.0, respectively. Two comparative experiments demonstrate the superior efficiency of the proposed GA over the STW and the UGA. The study implements AGV-encoding as a basis for a genetic algorithm that tackles the problem of scheduling AGVs. Conflict resolution strategies are presented, and a time window-based Dijkstra’s algorithm is employed to identify the shortest conflict-free route, facilitating efficient planning rooted in production scheduling. The study verifies the feasibility of the proposed approach by applying it to a real case.

Future research will investigate the impact of equipment maintenance and breakdowns on production scheduling and explore the use of advanced algorithms to enhance its efficiency.

## Figures and Tables

**Table 1:** Processing machines for each process and the time required.

Workpiece	Operation	Machine	Time(s)
1	1	2	8
	2	4	10
2	1	4	10
	2	3	14
3	1	1	20
4	1	3	6
	2	1	14
5	1	4	20

**Table 2:** Transportation time(s).

From/To	L/U	M_1_	M_2_	M_3_	M_4_
**L/U**	0	18	16	12	8
**M_1_**	18	0	14	14	18
**M_2_**	16	14	0	12	12
**M_3_**	12	14	12	0	16
**M_4_**	8	18	12	16	0

**Table 3:** Parameters and descriptions.

Parameter	Description
$J_{i}$	sets of workpieces tasks
$M_{k}$	sets of machines
$K_{v}$	sets of AGVs
$O_{ij}$	step j of task i
$S_{ijk}$	the start time of process c on machine k
$C_{ijk}$	the finish time of process $O_{ij}$ on machine k
$t_{ijk}$	the spending time of process $O_{ij}$ on machine k
$C_{i}$	the finish time of task i
$T_{ijv}$	AGV v is responsible for the transportation of process $O_{ij}$
$T_{{kk}^{\prime }}$	the travel time of the AGV from machine k to machine k’
$LT_{ijv}$	the starting node of AGV v in task $T_{ijv}$
$S^{\prime }T_{ijv}$	the start time of no-load transport in task $T_{ijv}$
$C^{\prime }T_{ijv}$	the finish time of no-load transport in task $T_{ijv}$
$ST_{ijv}$	the start time of load transport in task $T_{ijv}$
$CT_{ijv}$	the finish time of load transport in task $T_{ijv}$
$N_{s}$	set of nodes

**Table 4:** Results for cases where the t/p ratio is above 0.25.

Problem	t/p	LB	STW	UGA	Proposed Algorithm	Improvement
EX11	0.59	72	96	96	96	0
EX21	0.61	86	105	104	100	+4
EX31	0.59	81	105	105	102	+3
EX41	0.91	62	118	116	112	+4
EX51	0.85	60	89	87	87	0
EX61	0.78	96	120	121	118	+3
EX71	0.78	76	119	118	115	+3
EX81	0.58	146	169	152	161	−9
EX91	0.61	93	120	117	116	+1
EX101	0.55	124	153	150	147	+3
EX12	0.47	66	82	82	82	0
EX22	0.49	76	80	76	76	0
EX32	0.47	75	88	85	85	0
EX42	0.73	60	93	88	87	+1
EX52	0.68	54	69	69	69	0
EX62	0.54	86	100	98	100	−2
EX72	0.62	74	90	85	80	+5
EX82	0.46	140	151	142	151	−9
EX92	0.49	91	104	102	102	0
EX102	0.44	114	139	137	135	+2
EX13	0.52	64	84	84	84	0
EX23	0.54	82	86	86	86	0
EX33	0.51	77	86	86	86	0
EX43	0.8	58	95	91	90	+1
EX53	0.74	52	76	75	74	+1
EX63	0.54	88	104	104	103	+1
EX73	0.68	76	91	88	85	+3
EX83	0.5	142	153	143	153	−10
EX93	0.53	93	110	105	105	0
EX103	0.49	116	143	143	139	+4
EX14	0.74	68	108	103	103	0
EX24	0.77	84	116	113	108	+5
EX34	0.74	81	116	113	111	+2
EX44	1.14	62	126	126	126	0
EX54	1.06	56	99	97	97	0
EX64	0.78	90	120	123	120	+3
EX74	0.97	76	136	128	127	+1
EX84	0.72	148	163	163	163	0
EX94	0.76	91	125	123	120	+3
EX104	0.69	120	171	164	159	+5

**Table 5:** Results for cases where the t/p ratio is below 0.25.

Problem	t/p	LB	STW	UGA	Proposed Algorithm	Improvement
EX110	0.15	126	126	126	126	0
EX210	0.15	148	148	148	148	0
EX310	0.15	138	150	148	150	−2
EX410	0.15	112	121	119	119	0
EX510	0.21	102	102	102	102	0
EX610	0.16	163	186	186	186	0
EX710	0.19	137	137	137	137	0
EX810	0.14	271	292	271	292	−21
EX910	0.15	150	176	176	176	0
EX1010	0.14	218	238	236	242	−6
EX120	0.12	123	123	123	123	0
EX220	0.12	143	143	143	143	0
EX320	0.12	135	148	145	145	0
EX420	0.12	111	116	114	114	0
EX520	0.17	99	100	100	100	0
EX620	0.12	160	183	181	181	0
EX720	0.15	136	136	136	136	0
EX820	0.11	268	287	268	287	−19
EX920	0.12	150	174	173	173	0
EX1020	0.11	216	236	238	236	+2
EX130	0.13	122	122	122	122	0
EX230	0.13	146	146	146	146	0
EX330	0.13	136	149	146	146	0
EX430	0.13	110	116	114	114	0
EX530	0.18	98	99	99	99	0
EX630	0.14	161	184	182	182	0
EX730	0.17	137	137	137	137	0
EX830	0.13	269	288	270	288	−18
EX930	0.13	151	176	174	174	0
EX1030	0.12	217	237	241	237	+4
EX140	0.18	124	124	124	124	0
EX241	0.13	217	217	217	217	0
EX340	0.18	138	151	151	151	0
EX341	0.12	203	222	221	221	0
EX441	0.19	166	179	172	172	0
EX541	0.18	148	154	148	148	0
EX640	0.19	161	185	184	184	0
EX740	0.24	137	138	137	137	0
EX741	0.16	203	203	203	203	0
EX840	0.18	272	293	273	293	−20
EX940	0.19	149	177	175	175	0
EX1040	0.17	219	240	244	240	+4

## Data Availability

The data may be obtained from the authors upon reasonable request.
